# Supplementary material for: Content-rich biological network constructed by mining PubMed abstracts
Source: BMC Bioinformatics. 2004 Oct 8;5:147. doi: 10.1186/1471-2105-5-147 (PMC528731; doi:10.1186/1471-2105-5-147)
Supplement: Additional File 5 — The original Chilibot query results of the term "long-term potentiation (LTP)" and 22 other terms, limiting the latest references analyzed to the years 1990, 1995, 2000, and 2004. [file 1471-2105-5-147-S5.bz2 › chilibotAdditionalFile5/ltp1995/html/PKA_ZIF268.html]

 


 **PKA** and **ZIF268** 
  
Found 1 abstracts in PubMed,  **1 abstracts were retrieved and analyzed**.  


---

 Search Google  |
 PDF files only 
|  EDU domain only 

---

- Biochim Biophys Acta, 1995   **Differential regulation of primary response gene expression in skeletal muscle cells through multiple signal transduction pathways.** .
  One of the earliest cellular responses to growth factors is the rapid induction of primary response genes.
  One group of such genes was originally isolated as tetradecanoyl phorbol acetate TPA inducible sequences TIS genes from mouse 3T3 cells.
  Proteins encoded by the TIS genes include two transcription factors TIS8 also known as egr1 NGFIA **zif268** and TIS1 also known as NGFIB nur77 N10 .
  We have examined the inducibility of these two genes in a skeletal muscle cell line in response to agents that have been reported to block muscle differentiation.
  We report here that basic fibroblast growth factor bFGF induced the expression of both TIS1 and TIS8 in mouse C2C12cells.
  Both genes were also inducible by TPA while forskolin which activates the cAMP dependent pathway induced TIS1 but not TIS8.
  Down regulation of protein kinase C PKC activity by TPA pretreatment repressed the bFGF induction of TIS1 but had little effect on the bFGF stimulated expression of TIS8.
  Moreover, while both TPA and bFGF stimulated the hyperphosphorylation of c RAF and the activity of MAP kinase, TPA pretreatment failed to block RAF phosphorylation or the stimulation of MAP kinase activity by bFGF.
  Induction of the two TIS genes in skeletal myoblasts therefore appeared to be dependent to different extents on the activation of protein kinase A **PKA** , PKC and MAP kinase.
